# Transcriptional Abnormalities of Hamstring Muscle Contractures in Children with Cerebral Palsy

**DOI:** 10.1371/journal.pone.0040686

**Published:** 2012-08-16

**Authors:** Lucas R. Smith, Henry G. Chambers, Shankar Subramaniam, Richard L. Lieber

**Affiliations:** 1 Department of Bioengineering, University of California San Diego, La Jolla, California, United States of America; 2 Department of Orthopedic Surgery, Rady Children's Hospital, San Diego, California, United States of America; 3 Department of Orthopedic Surgery, University of California San Diego, La Jolla, California, United States of America; 4 Department of Veterans Affairs, Medical Center, San Diego, California, United States of America; University of Pittsburgh, United States of America

## Abstract

Cerebral palsy (CP) is an upper motor neuron disease that results in a spectrum of movement disorders. Secondary to the neurological lesion, muscles from patients with CP are often spastic and form debilitating contractures that limit range of motion and joint function. With no genetic component, the pathology of skeletal muscle in CP is a response to aberrant complex neurological input in ways that are not fully understood. This study was designed to gain further understanding of the skeletal muscle response in CP using transcriptional profiling correlated with functional measures to broadly investigate muscle adaptations leading to mechanical deficits.

Biospsies were obtained from both the gracilis and semitendinosus muscles from a cohort of patients with CP (n = 10) and typically developing patients (n = 10) undergoing surgery. Biopsies were obtained to define the unique expression profile of the contractures and passive mechanical testing was conducted to determine stiffness values in previously published work. Affymetrix HG-U133A 2.0 chips (n = 40) generated expression data, which was validated for selected transcripts using quantitative real-time PCR. Chips were clustered based on their expression and those from patients with CP clustered separately. Significant genes were determined conservatively based on the overlap of three summarization algorithms (n = 1,398). Significantly altered genes were analyzed for over-representation among gene ontologies and muscle specific networks.

The majority of altered transcripts were related to increased extracellular matrix expression in CP and a decrease in metabolism and ubiquitin ligase activity. The increase in extracellular matrix products was correlated with mechanical measures demonstrating the importance in disability. These data lay a framework for further studies and development of novel therapies.

## Introduction

CP (CP) is a movement disorder caused by an upper motor neuron (UMN) lesion in the developing brain [1]. There are a range of prenatal, perinatal, and postnatal causes of CP and it is often associated with periventricular leukomalacia [2]. CP covers a spectrum of severities and is the most common childhood movement disorder with a prevalence of 3.6 cases per 1000 in the US that has not decreased with medical advances [3]. While the UMN lesion that initiates CP is non-progressive, many secondary changes occur within the musculoskeletal system that are progressive and debilitating [4]. Among the hallmarks of CP is muscle spasticity, in which the muscle contracts in a velocity dependent resistance to stretch that results, in part, from reduced inhibition of the stretch reflex [5]. While the disability that results from spasticity is variable [6], [7] patients with spastic CP may also develop muscle contractures secondary to the lesion. Fixed muscle contractures represent a unique muscle adaptation in which muscles detrimentally limit the range of motion around a joint without being activated. These muscle contractures limit mobility, may be painful, and represent a major disability among those affected by CP or anyone with an UMN lesion [8].

There are a variety of treatments designed to inhibit muscle activity in CP and prevent contracture formation. Physical therapy techniques, oral muscle relaxants, intrathecal placement of medication (baclofen), chemical neurectomies with phenol or alcohol, chemodenervation using neurotoxins, and surgical neurectomies have all been employed to decrease spasticity in children with CP [9]. However, despite best clinical practices, contractures still develop and often require surgery to correct [10]. It should also be noted that all of these therapies reduce muscle strength in a condition in which strength is already compromised. Clearly current therapies are not ideal.

There are no known genetic defects in patients with CP and their muscles, as it is a direct consequence of the UMN lesion [11]. Although skeletal muscle is known to be highly adaptive in response to neurological input, muscle contractures that develop are part of an adaptive mechanism that is not fully understood. Contracture does not develop in animal models of increased muscle use, which could be present from decreased motor neuron inhibition, or even decreased muscle use, which could result in decreased functionality [4]. Indeed UMN contractures are not readily reproducible in animal models, thus necessitating research on human subjects [12]. The underlying transcriptional alterations have important consequences in the development of increased passive mechanical properties and pathologic contracture. Understanding the precise nature of transcripts differential regulation can delineate the mechanisms that accompany contracture, including but not limited to increased passive tension.

Previous research demonstrated that muscle stiffness in contracture is independent of active muscle contraction [13], [14]. Recent mechanical measurements of biopsies from pediatric hamstring muscles indicate that the increased muscle stiffness is due to alterations in extracellular matrix (ECM) rather than the stiffness of muscle fibers themselves [15]. Multiple studies have also shown an increase in sarcomere length of muscle in contracture, demonstrating contractured muscle experiences high intrinsic strain [16] due to dramatic, but unknown structural alterations. These results suggests a decrease in the serial sarcomere number despite conflicting evidence as to whether muscle fascicle length decreases [17]–[20]. Studies have shown that muscle and muscle fiber cross sectional area are reduced, which decreases force production, and even that the remaining muscle has decreased force generating capacity [21]–[23]. These mechanical and architectural changes in muscle implicate a disruption of the biological components involved in myogenesis, force generation, force transmission, extracellular matrix maintenance, and perhaps additional pathways. Recent microarray data from the upper extremity supports the assertion that CP muscle is altered transcriptionally and that in addition to the pathways listed above, neuromuscular junction activity, excitation-contraction coupling, and energy metabolism are also deranged [24].

As a purely adaptive muscle disorder, contractures are believed to have an altered transcriptional profile. The current study has taken advantage of a large surgical population of both children with CP and typically developing children to conduct a robust microarray analysis correlated to mechanical parameters. Our previous study was limited by a very small control subject population which was not age matched, (N = 2) [24], and microarray studies in humans subjects generally require larger sample sizes to identify differences due to the higher variability present in human tissues compared to most inbred animal strains. Additionally the same biopsies reported here have been used to collect mechanical data that was recently published, allowing the comparison of our transcriptional data to functional parameters [15]. We also took advantage of recent additions of muscle specific gene ontologies and muscle specific gene networks to probe the muscle and compare the pathology to microarrays from other published muscle conditions ([25]; Smith et al. in press). A mechanistic understanding of muscle adaptation to contracture may lead to discovery of possible therapeutic targets that can delay or even reverse the debilitating effects of CP or other UMN lesions.

## Results

For this transcriptional study, a cohort of 20 subjects were recruited. 10 patients with cerebral palsy were undergoing hamstring lengthening surgery, making biopsies of gracilis and semitendinosus accessible. The disease parameters of these subjects are listed in Table 1. Gracilis and semitendinosus are synergistic muscles that both create a knee flexion moment, and thus their surgical release facilitates knee extension. Surgery is required because both muscles studied are in the state of pathologic muscle contracture. As control subjects, 10 typically developing pediatric patients who were approximately age matched were included, who were undergoing ACL reconstruction surgery with a hamstring autograft that made gracilis and semitendinosus muscles accessible. A separate microarray was used for each muscle biopsy, resulting in a total of 40 microarrays.

**Table 1:** Subject parameters.

Cerebral Palsy Subjects						Typically Developing Subjects		
Subject ID	Sex	Age (years)	Region	GMFCS	Popliteal Angle	Subject ID	Sex	Age (years)
8	M	16	Q	V	90	1	M	14
9	M	11	H	II	110	2	F	16
30	M	4	D	II	125	5	F	13
32	F	8	D	II	95	6	F	15
34	M	15	D	II	120	10	M	15
35	M	6	Q	V	130	12	M	16
37	M	9	H	II	95	18	F	17
38	M	9	D	II	100	28	M	15
39	M	15	D	II	120	31	M	14
40	M	10	D	II	120	33	M	13

The parameters for each subject is specified. Region indicates the regions of the body effected: (Q) quadriplegic, (H) hemiplegic, and (D) Diplegic. Gross Motor Function Classification System (GMFCS) score is a functional parameter ranging from I (least severe) to V (most severe). Popliteal angle is a measure of knee extension and represents the maximum angle of the upper leg to lower leg when the hip is flexed at 90°.

### Significantly altered genes

Among the 22,283 probesets on the microarray, 13,787 were considered present for further analysis. Of those 1,398 genes were identified as significantly different in CP (2,836 for Microarray Suite Version 5.0 (MAS5), 3,954 for Robust Multiarray Analysis (RMA), and 4,009 for GC-RMA; Table S1). Of these genes, 533 had expression increased in CP while 865 genes had expression decreased. The 2×2 ANOVA yielded only 3 genes (*MAB21L1*, *SIM1*, and *EN1*) with unknown roles in muscle as significantly different between muscles, demonstrating that both muscles have similar expression profiles. There were also no genes that produced a significant interaction between muscle type and disease state, indicating that both muscles undergo similar changes in CP.

### Condition tree clustering

**Figure 1:**
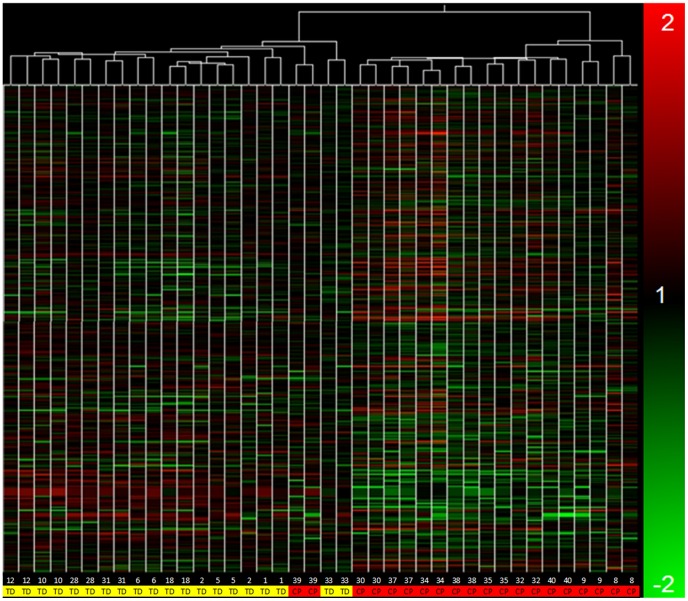
Condition tree from Pearson correlation clustering algorithm. Individual genes are colored according to expression ratio. CP samples cluster separately from typically developing controls, except for one subject. Both subject sample being clustered together indicates relatively little variability between gracilis and semitendinosus biopsies from the same subject. Table contains Subject ID in row 1 and either typically developing (TD) or CP (CP) in row 2.

The condition tree was conducted based on all present genes on the microarray and resulted in a clustering of CP subject biopsies distinct from typically developing subject biopsies, with the exception of one mild CP subject (Figure 1). The condition tree also shows that biopsies from the two muscles of the same patient cluster together. This result indicates that the two hamstring muscles, gracilis and semitendinosus, have less variability within a subject than the variability between subjects with the same condition. With a large sample size there was an expectation that biopsies from patients with similar clinical severity scores would cluster together. This trend was not observed in the data.

### Quantitative Real Time PCR

**Figure 2:**
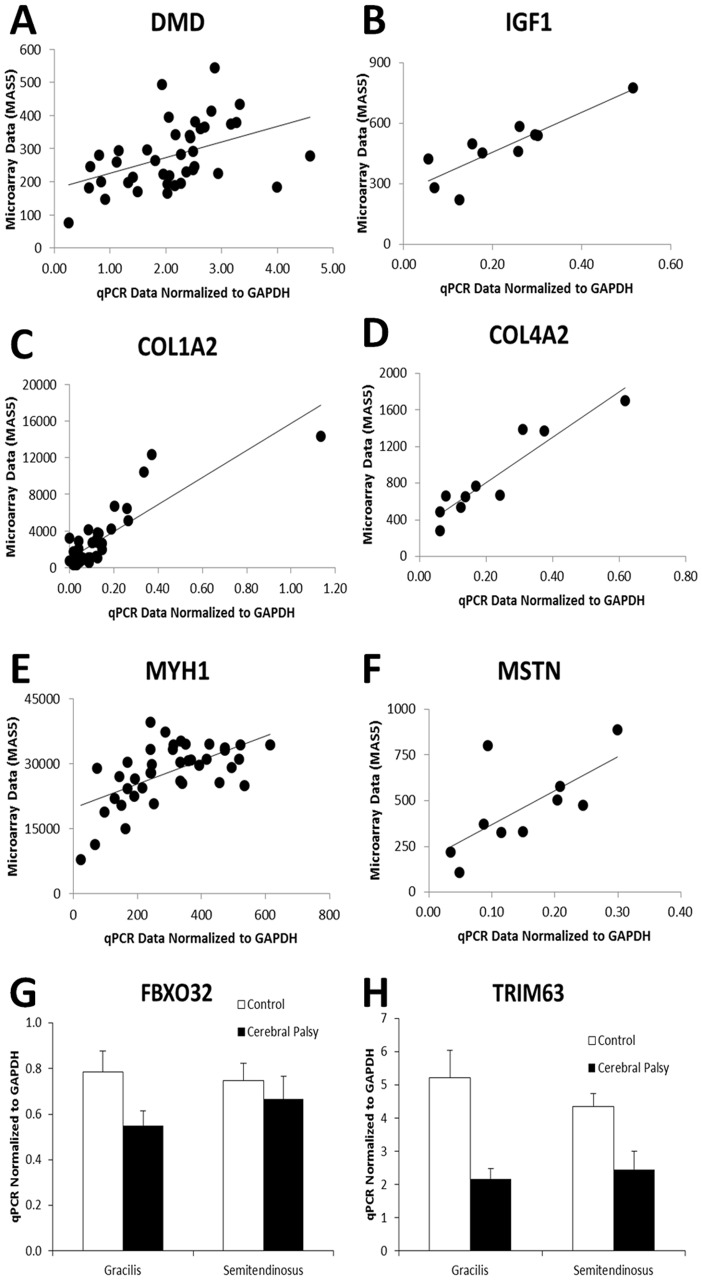
Comparison of quantitative real-time PCR data to microarray data. The microarray data from MAS5 summarization algorithm is used. Each gene in A–F has a significant correlation (p<0.05). (G) atrogin-1 (FBXO32) and (H) MURF-1 (TRIM63) are quantified based on disease state and muscle as they are not present on the microarray.

As a quality control measure, qRT-PCR was performed on a subset of relevant genes, 2 significantly up-regulated, 2 not significantly altered, 2 significantly down regulated, and 2 genes of interest not present on the microarray. We chose these representative genes since they are commonly studied in muscle physiology. Comparison of qRT-PCR to microarray datasets revealed a significant correlation (p<0.05) in all 6 genes examined (Figure 2A–F). qRT-PCR was also conducted on 2 genes of interest involved in the muscle atrophic process that do not have corresponding probesets on the microarray that had expression ratios of (0.48) *TRIM63* and (0.79) *FBXO32*. *TRIM63* expression had a significant main effect of CP (p<0.01) (Figure 2G–H).

### Categorical analysis

Secondary analysis was performed using predefined gene sets for Gene Ontology allowing the categorization of significantly altered genes in CP. There were 87 gene ontologies over-represented from the list of significantly genes up-regulated in CP (Table S2, Table 2). Of note are a large portion of extracellular matrix ontologies as well as those for calcium ion binding and cytoskeleton ontologies. Categories discussed are presented in Table 2. Among genes that were down-regulated in CP, 85 ontologies were significantly over-represented (Table S2, Table 2). These ontologies primarily fell into the categories of metabolic processes and ubiquitin related pathways and also interestingly included skeletal muscle contraction.

**Table 2:** Highlighted gene ontologies significantly over-represented in genes significantly altered in CP.

Gene Ontology	GO#	Category	Observed	Expected	p-value
*Biological Process*	*Increased in CP*				
extracellular matrix organization	0030198	86	20	3.57	1.55e-07
collagen metabolic process	0032963	38	8	1.58	0.0044
actin cytoskeleton organization	0030036	213	22	8.85	0.0044
*Molecular Function*					
calcium ion binding	0005509	680	64	28.22	3.91e-08
collagen binding	0005518	32	8	1.33	0.0005
growth factor binding	0019838	97	14	4.02	0.0006
*Cellular Component*					
collagen	0005581	31	12	1.31	4.60e-08
cytoskeleton	0005856	1008	75	42.73	1.58e-05
basal lamina	0005605	15	6	0.64	0.0003
*Biological Process*	*Decreased in CP*				
ubiquitin-dependent protein catabolic process	0006511	190	33	12.18	9.76e-06
glucose metabolic process	0006006	116	19	7.43	0.0041
skeletal muscle contraction	0003009	15	6	0.96	0.0073
*Molecular Function*					
ligase activity	0016874	281	43	18.12	6.48e-06
ubiquitin-protein ligase activity	0004842	107	23	6.90	1.39e-05
zinc ion binding	0008270	1447	128	93.32	0.0028
*Cellular Component*					
proteasome complex	0000502	54	11	3.35	0.0047
cis-Golgi network	0005801	12	5	0.74	0.0056
nuclear lumen	0031981	1203	101	74.65	0.0081

(Category) is the number of genes on the reference list or in this case on the microarray that fall under the ontology. (Observed) is the number of genes on either the significantly up or down-regulated in CP list. (Expected) is the number of genes expected to be in the significantly altered list based on the relative sizes of lists.

### Network analysis

**Figure 3:**
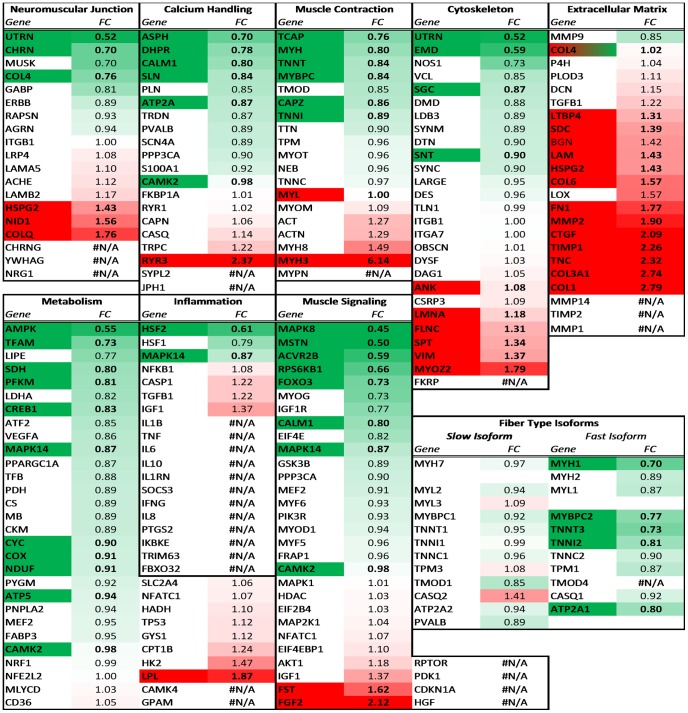
Heatmap of functional muscle gene networks. Heatmap based on expression ratio and separated by Entrez gene symbols are used for individual entities with the exception of gene families found in Table S3 in which case geometric means of multiple genes determine expression ratio. Gene symbols found to be significantly different in CP are colored based on direction of regulation. Gene families are colored if any individual gene in the family is significantly altered in CP. Genes with N/A were either not present on the chip or did not have expression to qualify as present in analysis.

We recently created networks of genes related to muscle function to enable detailed investigation of muscle-specific gene expression. A heatmap, using expression ratios, is created with genes listed in their 9 respective functions with genes in those determined as significantly different in CP according to previous analysis (Figure 3). Some genes appear in multiple functions and some genes are listed within gene complexes (Table S3).

Most of the networks had a selection of genes that were both up and down regulated in CP. These include: the neuromuscular junction, excitation contraction coupling, cytoskeleton elements, and muscle signaling. Other networks had transcripts that were altered in a more uniform manner. Most strikingly, the extracellular matrix had 14 out of 20 transcripts with increased transcription. On the other hand muscle metabolic factors had 11 out of 37 transcripts down regulated and only one up regulated. Unfortunately only 7 of the inflammatory markers were present and available for analysis on the microarray. Some proteins function with a different isoform in fast or slow muscle that is transcribed from a separate gene. There was no significant change in expression among the slow isoforms, however 5 out of the 11 transcripts for fast isoforms were significantly down regulated. This indicates a relative shift to slow fibers that corresponds to the myosin heavy chain protein content of the muscles [15].

### mRNA correlations to passive stiffness

**Figure 4:**
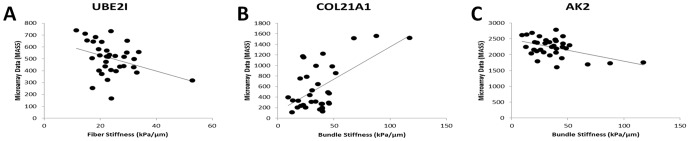
Correlation between transcript levels and stiffness. Examples of significant correlation (p<0.05) between mRNA expression levels and passive mechanical stiffness measurements. (A) ubiquitin-conjugating enzyme E2I (UBE2I) has a negative correlation with fiber stiffness. (B) Collagen XXI alpha I (COL21A1) has a positive correlation with fiber bundle stiffness. (C) adenylate kinase 2 (AK2) and a mitochondrial intermembrane transcript has a negative correlation with bundle stiffness.

A unique aspect of this study was the corresponding mechanical measurements from the same muscle biopsies in 17 of the 20 subjects. Individual fibers were isolated and mechanically tested to determine stiffness [15]. Fiber bundles, consisting of a group of approximately 20 fibers and their constituent extracellular matrix, were tested in the same way. Mechanical stiffness for fibers and fiber bundles was correlated with each of the significant genes in CP. 27 genes had a significant (p<0.05) positive correlation with fiber stiffness and 50 genes had a significant negative correlation (Table 3A, 3B; Figure 4; Table S4). These gene lists were used for categorical gene ontology analysis with the total significantly altered in CP gene list as the reference set to determine which categories were over-represented (Table S5). From the genes positively correlated with fiber stiffness there were no categories significantly up-regulated. However among the negatively correlated genes, 29 ontologies were significantly (p<0.05) over-represented, with most ontologies related to ubiquitin ligase activity. This suggests that when genes in the ubiquitin protease system are most active muscle fibers lose mechanical stiffness. When fiber bundle mechanics are considered, 141 genes had a positive and 95 genes a negative significant correlations with stiffness values (Table 3C, 3D). There were 36 ontologies significantly over-represented with a positive correlation to bundle stiffness. These consisted almost exclusively of ontologies related to the extracellular matrix, indicating the matrix plays an important role in the stiffness of fiber bundles. Only 2 ontologies were significantly over-represented among genes negatively correlated with bundle stiffness. Those related to mitochondrial structure, suggesting a relationship between muscle stiffness and energy production.

**Table 3:** Gene ontology of transcripts correlated with stiffness.

Gene Ontology	Observed	Expected	p-value
*Biological Process (selection from 21)*	*Positive Bundle Stiffness Correlation*		
extracellular matrix organization	13	2.25	5.54E-06
collagen fibril organization	8	1.07	7.53E-05
collagen biosynthetic process	5	0.54	1.40E-03
*Molecular Function (selection from 5)*			
extracellular matrix structural constituent	12	2.25	2.58E-05
growth factor binding	8	2.25	2.56E-02
proteoglycan binding	3	0.32	2.60E-02
*Cellular Component (selection from 10)*			
proteinaceous extracellular matrix	22	5.46	1.65E-07
fibrillar collagen	6	0.75	2.00E-04
basement membrane	8	1.93	4.70E-03
*Cellular Component*	*Negative Bundle Stiffness Correlation*		
mitochondrial intermembrane space	4	0.36	6.70E-03
organelle envelope lumen	4	0.43	1.00E-02
*Biological Process (selection from 20)*	*Negative Fiber Stiffness Correlation*		
ubiquitin-dependent protein catabolic process	7	1.34	2.54E-02
cellular protein catabolic process	9	2.89	3.18E-02
proteasomal protein catabolic process	4	0.60	3.81E-02
*Cellular Component (selection from 9)*			
nucleus	28	16.61	1.44E-02
proteasome complex	4	0.48	2.16E-02
membrane-bounded organelle	35	25.73	2.76E-02

Gene ontologies that were over-represented among genes that had a significant correlation (p<0.05) with either fiber or fiber bundle passive stiffness measurements. (Category) is the number of genes on the reference list or in this case significantly altered in CP. (Observed) is the number of genes significantly correlated with mechanical stiffness that fall into the category. (Expected) is the number of genes expected to be in the significantly altered list based on the relative sizes of lists.

## Discussion

The objective of this study was to describe the transcriptional adaptations that occur in skeletal muscle of patients with CP and incorporate functional data to determine the cellular mechanisms that drive the muscle pathology secondary to the upper motor neuron lesion. This research has seen little prior work, as there is no commonly accepted animal model necessitating the challenges of direct human research [12]. Without any genetic defect in the muscle, it is clear that the pathology has a large transcriptional component across many genes (Figure 1). The large number of altered transcripts fall into a variety of gene ontologies and biological pathways, as well as important functional networks within skeletal muscle. Some of these vital muscle systems such as the extracellular matrix have a correlation between transcript levels and tissue stiffness.

The condition tree clustering demonstrates the difference between the transcriptional profile of muscles from patients with CP or typically developing (Figure 1). This separation was nearly 100% as only a single CP patient clustered more closely with the controls. It should be noted that this was one of the more mild cases of CP and the highest popliteal angle of the entire group (120°, least amount of contracture). The clustering algorithm did not, however, group the CP muscle by the clinical severity scores, age, or even muscle. The patients themselves had both biopsies clustered together consistently indicated that the variation from subject to subject is higher than that between muscles of the same subject. This finding was described in a previous study using multiple muscles from a single subject [24].

### Categorical analysis

To determine the various functions that are altered in CP we first established a list of genes with altered transcription. We sought to narrow our results by using a more stringent analysis that uses the congruence of three summarization algorithms as well as a restrictive false discovery rate of 1%. This still produced a large gene list due to the large sample size, but we chose not to implement a fold change cutoff as this assumes an arbitrary level of change is required to be functionally important [26]. We used categorical analysis to determine altered functional gene categories in CP.

Among the up-regulated transcripts there were a total of 87 gene ontologies over-represented, but they generally fell into only a few categories. The increase in extracellular matrix transcripts has been well documented in muscle from CP [24], [27], [28]. However the structure of the extracellular matrix increases is not well known. This study shows how extensive the increase in extracellular matrix is with categories ranging from fibrillar collagen, basal lamina and even collagen metabolic process. Increased structural categories continue through the cell with ontologies including integrin binding, cytoskeletal binding protein, and cytoskeleton. This could have an effect on individual fiber stiffness, but the stiffness of fibers in CP is inconsistent [15], [29]. There were ontologies important in muscle function that had over-representation as well with calcium ion binding. A disruption of calcium handling had been suggested previously, where *PARV* was drastically altered in CP muscle [24], however here *PARV* was unchanged. The impact of growth factor binding is also crucial in skeletal muscle, with many of the transcripts being IGF binding proteins known to be important in skeletal muscle.

Contrastingly, there were 62 ontologies that were over-represented in down-regulated genes. The most extensive of these were related to metabolic processes and ubiquitin ligases. Skeletal muscle is a very metabolically active tissue and these results suggest that, despite spasticity, the muscle has less metabolic machinery. This decrease was also seen in the previous transcriptional study in CP, however this was accompanied by a shift to faster fiber types, which was not the case here [24]. The ubiquitin ligase role is surprising here as it is generally accepted that muscle in CP has decreased mass [21], [30]. This result suggests that decreased muscle mass is not due to active muscle degradation. The obvious importance of the skeletal muscle contraction ontology is also represented. Together these results imply that muscle protein turnover is decreased in CP.

### Network analysis

Categorical analysis provides an avenue to analyze vast gene lists into manageable pieces. However, we also sought to investigate more deeply into networks of genes critical to muscle function using a recently established gene networks (Smith et al., in press). As CP is neurological in origin, the neuromuscular junction could be altered and has indeed been shown to have a disorganized nature in CP [31]. The results did not show overwhelming changes in the neuromuscular junction, the acetylcholine receptor (*CHRN*) was down-regulated. *CHRN* is dramatically up-regulated when muscle is denervated [32], which our results clearly show is not the case in CP muscle. Most of the changes were associated with extracellular matrix proteins localized to the neuromuscular junction. However, unlike the majority of extracellular matrix transcripts *COL4* was down-regulated as was *UTRN* an important link to the extracellular matrix of the neuromuscular junction. It is unclear how these adaptations change the function of the neuromuscular junction, but it does support evidence for disorganization [31].

Excitation-contraction coupling has been largely unexplored in CP, but has been shown to be altered and is also the target of therapeutic intervention [24], [33]. The transcripts altered in this study were unique however, and primarily down-regulated in CP. Genes such as *ASPH* and *DHPR* which have a role in activating the -ryanodine receptor are down-regulated. As are mechanisms for pumping calcium back in to the sarcoplasmic reticulum with *ATP2A* and its regulator *SLN*. Further, a decrease in transcripts of calcium binding proteins of *CALM1* and downstream *CAMK2* suggest that there is less calcium cycling in CP muscle. The only up-regulated transcript was *RYR3*, which is a ryanodine receptor expressed in immature muscle, and reinforces the theme that there are a number of immature transcripts in these muscles.

That theme is also observed in the contractile transcripts of skeletal muscle. The only up-regulated genes are immature isoforms embryonic myosin heavy chain (*MYH3*) and embryonic myosin light chain (*MYL4*). Many of the contractile elements have well defined isoforms that have genes expressed in either fast or slow muscle [34]. Many of the down-regulated transcripts are isoforms of the fast isoforms, which will be discussed with respect to fiber type. Interestingly, both thin filament Z-disc capping protein *CAPZ* and titin filament Z-disc capping protein *TCAP* are down-regulated in CP. This could lead to Z-disc disorganization and *TCAP* itself is associated with destabilization in limb girdle muscular dystrophy 2G [35].

The force generated in the sarcomere is transmitted through the cytoskeleton to the cell periphery, but the effects in CP have been largely unexplored. Our results are somewhat difficult to interpret with many genes both up and down-regulated. Of those associated with the dystroglycan complex, sarcoglycans (*SGC*) and snytrophins (*SNT*) as well as *UTRN* are down-regulated. This is while many crosslinking transcripts of the cytoskeleton are up-regulated (ankyrin (*ANK*), sprectrin (*SPT*), filamin (*FLNC*)) along with an important connector to the Z-disc (*MYOZ2*). What role these increased cytoskeletal filament connections may play is unknown, but they did not lead to increased fiber stiffness [15]. The cytoskeletal connection to the nucleus is interesting as *EMD* and *LMNA*, which both lead to Dreifuss-Emery muscular dystrophy when absent are not co-regulated with *EMD* decreased and *LMNA* increased [36], [37]. One aspect that is consistent is that *VIM* is up-regulated, which is the primary immature muscle intermediate filament that is replaced with desmin (*DES*) during development.

The force generated in the cell is ultimately transferred to the extracellular matrix, which is significantly altered in various analyses here as well as numerous other studies of CP [24], [27], [28]. This alteration is nearly uniformly up-regulated, with the exception of the *COL4* isoforms in the neuromuscular junction previously described. It is important to note that the increase includes many categories, fibrillar collagens, laminar collagens, proteoglycans, matrix metalloproteinases, matrix metalloproteinase inhibitors, and extracellular matrix growth factors. This uniform increase does not permit speculation on how the extracellular matrix may be prolific, yet disorganized as speculated in CP muscle [28]. With all the increase in extracellular matrix components it would be expected that *TGFB1*, an important fibrosis signal in muscle, would be increased [38]. Although it did have higher expression in CP it was not significant and shows that ECM alterations can occur independent of a large *TGFB1* autocrine increase and may be more closely tied to TGFβ activation locally.

Another network with broad regulation was metabolic transcripts, which were down-regulated in CP. This was observed in gene ontologies, pathways, as well as previous studies [24]. The fold change values on many of the transcripts were relatively low. It should be noted that this change occurred despite a decrease in fast muscle isoforms that have fewer mitochondria present. The only increased transcript was *LPL* associated with fat metabolism that is more prevalent in slow muscle. Despite an increase in non-voluntary muscle contractions associated with spasticity [39], the muscle is not producing more metabolic machinery. This could be a result of the muscle being in the state of contracture and thus have decreased functionality which contributes to disuse [40].

While the state of damage within a static muscle contracture is relatively unknown, it is known that inflammation is present within damaged muscle [41]. Wound healing and inflammatory pathways were up-regulated in categorical analysis, but unfortunately few inflammatory transcripts were able to be analyzed in this study. Of those that were altered were p38 (*MAPK14*) and a heat-shock transcription factor (*HSF2*). The role of muscle damage in pathologic CP muscle is not discernable from these data.

The critical aspect of muscle growth is a complicated system of many genes and viewed the lack of muscle growth is considered the primary cause of contracture [42]–[44]. However there are many transcripts altered to induce muscle growth including down-regulation of myostatin (*MSTN*) a critical muscle growth inhibitor, its receptor (*ACVR2B*), and up-regulation of natural inhibitor (*FST*). *FGF2* also plays a role in stimulating muscle growth and differentiation [45]. The muscle atrogene program is also decreased with a drop in MURF1 (*TRIM63*) and its transcription factor *FOXO* [46]. This *MSTN* signal is in contrast to the previous study in CP muscle and is a candidate for being responsible for blocking the growth signal in these muscles. The question of what limits muscle growth in CP is not apparent from these results.

The role of fiber type has been discussed in regard to multiple networks. The results here show clearly that while slow isoform levels remain unchanged many fast isoforms have significant decreases in transcription. The role of fiber type in upper motor neuron lesions has been inconsistent [47], [48], but these results imply a proportional shift to slower muscle. This can be important functionally, but is also very important in terms of a transcriptional study. Many of the changes observed could be the result in transcriptional changes from fast to slow muscle, such as the decrease in glycolytic transcripts. However it is clear that the pathology of muscle in CP is not purely a secondary effect of a shift in fiber type.

### mRNA correlations to passive stiffness

Having mechanical stiffness measurements for both the muscle fibers and the muscle fiber bundles with their extracellular matrix was a unique aspect of this study. There is literature to support the role of the giant protein titin (*TTN*) in being the major contributor to passive tension of individual fibers [49]. When considering the muscle bundle much of the passive stiffness is believed to arise from collagen, especially at larger sarcomere lengths [50]. Certainly many transcriptional factors could contribute to the production of these important proteins or other proteins that have a direct impact on the passive mechanical stiffness. Indeed 77 genes were correlated with fiber stiffness and 236 with bundles stiffness. To determine the fundamental nature of these transcripts we again used categorical analysis. It should be noted that since only significantly altered genes were considered that was the gene set used as a reference set, meaning correlated genes had to be enriched beyond the significant gene list be over-represented.

For individual fiber there were actually no ontologies that had a significant positive correlation with stiffness. However many ontologies were associated with decreasing fiber stiffness, the vast majority of which were related to ubiquitin ligases. This supports the idea that as ubiquitin ligases become active and begin degrading the muscle filaments, particularly titin, causing a loss of passive stiffness [51].

When bundle stiffness is considered there are many more ontologies over-represented (Table 3). Fittingly the most prevalent ontologies are related to extracellular matrix, however there are many more transcripts than fibrillar collagens thought to provide the passive stiffness to bundles [52]. For example one of the most highly correlated genes was *COL21A1*, which is a fibril associated collagen that is found with collagen I and serves to maintain the integrity of the extracellular matrix. This illustrates the complex nature and the many factors that lead to tissue stiffness and that could be contributing to fibrosis in CP. Additional ontologies were over-represented including immune system process. This is an indicator of damage in bundles that are stiffer and supports the claim that muscle tissue damage leads to fibrosis in CP. Conversely there were only two ontologies negatively correlated to bundle stiffness, related to mitochondria. It is difficult to speculate in too much detail from this small data set, but this suggests that more metabolically active tissue, with increased mitochondria, is more compliant. Perhaps this is simply a secondary effect of decreased area fraction in myofilaments occupied by mitochondria.

### Comparison to upper extremity

This study is similar to one conducted in the upper extremity of muscle from patients with CP [24]. There are some important differences in that the current study has a much larger sample size, especially in terms of controls (10 in this study compared to 2 in the previous). Additionally the typically developing control subjects in the current study were injured months prior to surgery and thus did not have recent acute trauma as a confounding factor. Regardless, we expected to see many similar results between the two studies. However this was not the case with only 19 genes significantly altered in the same direction between the two studies (Table S6). A possible explanation for this is the change in fiber type, which was opposite in the studies, was driving many of the transcriptional changes. Fast fibers have a more extensive calcium cycling apparatus thus offering an explanation other than a disruption of typical calcium handling in CP [24]. It is still important to look at the similarities between the studies. The obvious similarity is an increase in extracellular matrix in both upper and lower extremity contracture, shown with ontology analysis of genes altered in both studies. This is important in placing fibrosis as a consistent property of muscle contractures in CP. It should also be noted that both studies had an increase in genes related to immature muscle, another possible hallmark of muscle in a contractured state.

### Implications

Despite CP being a spectrum disorder that is non-homogenous in our subjects there are many common attributes [53]. This study is valuable in highlighting many functions of skeletal muscle that are disrupted in contractures caused by CP and detailing the gene transcripts involved. It does not directly answer which programs are inducing contracture or contributing to the lack of muscle growth. However the most dramatic changes were seen in the drastic increases in extracellular matrix, which could blunt the intracellular growth signals observed. Indeed it has been shown that the extracellular stiffness can modulate skeletal muscle satellite cells proliferation and differentiation [54], [55]. Further matrix digestion has been shown to have a positive effect on muscle growth through satellite cell activation [56]. Combining previous knowledge with this study establishes the extracellular matrix as a novel treatment target for CP.

### Summary

Skeletal muscle undergoes significant transcriptional alterations secondary to upper motor neuron lesion in CP in which we have identified many transcripts. These genes fall into several ontologies with increases extracellular matrix components along with decreases in metabolic, and muscle degradation systems. Muscle specific network analysis recapitulates the increase in extracellular matrix components along with an increase in muscle molecules signaling for muscle growth. These are overlaid on a decrease in fast muscle isoform transcripts and an increase in immature muscle isoforms. The increases in extracellular matrix were seen to be associated with an increase in the passive stiffness of the muscle tissue and one of the few components consistent with previous transcriptional studies into CP muscle. This work will assist future research into CP muscle and aid the design of novel therapies for these patients.

## Materials and Methods

### Muscle Biopsy Collection

Ethical approval for this study conformed to the standards of the Declaration of Helsinki and was approved by the Institutional Review Board at the University of California, San Diego Human Research Protection Program. Age appropriate assent from the patient as well as consent of the parent or guardian was obtained. After obtaining consent, subjects with spastic CP (n = 10) were recruited into the study based on undergoing distal hamstring lengthening surgery involving both the gracilis and semitendinosus muscles such that 2 muscle biopsies could be acquired per subject. “Control” subjects (n = 10) were pediatric patients undergoing ACL (anterior cruciate ligament) reconstructive surgery of the knee with hamstring autograft using gracilis and semitendinosus tendons that were excised along with a distal portion of the muscle that was obtained prior to trimming of the tendon. Control patients did not have any neuromuscular disorders and were ambulatory prior to surgery suggesting no damage to the hamstring muscle from the injury. Their index injury occurred at least 6 weeks before their surgery. However, because they were having surgery to repair a torn ligament we acknowledge that these are not truly normal muscles. However, given the ethical constraints associated with taking muscle biopsies, we believe that this is the best possible comparison group that can be envisioned. Patients with CP had developed a fixed contracture requiring surgery and were classified based on the clinical measures of Gross Motor Function Classification System [57], popliteal angle, and limb(s) affected. Patients had not received any neurotoxin injection or previous surgical lengthening within the 2 years prior to surgery. All muscle biopsies (n = 40) were snap frozen in liquid nitrogen (−159°C) within ∼1 minute of excision and stored at −80°C.

### RNA extraction

RNA was extracted using a combination of standard Trizol (Invitrogen, Carlsbad, CA) and RNeasy (Qiagen, Valencia, CA) protocols. Briefly, approximately 30 mg of frozen muscle tissue was homogenized using approximately 50 mg of RNase free 0.5 mm zirconium oxide beads (Next Advance, Averill Park, NY) in 0.5 ml Trizol using a Bullet Blender (Next Advance, Averill Park, NY). 0.1 ml of chloroform was added to the solution, then vigorously vortexed for 15 seconds, then centrifuged at 4°C for 15 min. The upper aqueous layer was removed and mixed with an equal volume of 70% ethanol before being added to the RNeasy spin column. The column was washed and then incubated with RNAse-free DNAse (Qiagen) for 15 minutes and then washed again three times prior being eluted as described in the manufacturer's protocol. RNA concentration was determined by absorbance at 260 nm, and the 260 nm-to-280 nm absorbance ratios were calculated to define RNA purity.

### Microarray processing

Affymetrix microarrays (HG-U133A 2.0; Affymetrix, Santa Clara, CA) were used for each individual muscle biopsy (n = 40 chips). RNA processing including cDNA synthesis, cDNA labeling, microarray hybridization, microarray scanning, and stringent quality control measures were performed by the Gene Chip Core at the Department of Veterans Affairs San Diego Health Care System, (San Diego, CA). The raw data are available (.cel files) at the Gene Expression Omnibus (GEO) under accession number GSE31243.

### Quantitative real time PCR

Quantitative real-time PCR (qRT-PCR) was conducted to validate the expression levels of select genes (*DMD*, *COL1A2*, *MSTN*, *IGF1*, *COL4A2*, and *MYH1*) and also to provide expression values for two transcripts of interest not present on the microarray (*FBXO32* and *TRIM63*). The same RNA extracted for microarray analysis was used for qRT-PCR. Isolated RNA was diluted 1∶5 with DNase/RNase free water (Invitrogen) and 1 µl of each sample was reverse transcribed using standard protocols (Superscript III; Invitrogen). cDNA was amplified with the Cepheid SmartCycle (Sunnyvale, CA) with primers designed specific to each gene of interest (Table S7) using nBLAST and Oligo (version 6.6; Molecular Biology Insights, Cascade, CO). Each sample was run in triplicate along a standard curve. The PCR reaction tube contained 1×PCR buffer, 2 mM MgCl_2_ (Invitrogen), 0.2 mM sense and antisense primers, 0.2 mM dNTP, 0.2×SYBR green, and 1 U of platinum Taq polymerase (Invitrogen). Amplification conditions included an initial hold at 95°C for 2 minutes with 40 cycles of denaturing at 95°C for 15 seconds, followed by annealing and extension phases adjusted for each transcript. Success of each the reaction was determined based on observation of a single reaction product on an agarose gel and a single peak on the DNA melting temperature curve determined after the 40 cycles. The results of qRT-PCR were expressed using a standard curve method with the “cycles to threshold” values represented the number of PCR cycles at which the SYBR green signal was increased above threshold. The triplicate measures were normalized to the housekeeping gene *GAPDH* and then averaged. qRT-PCR data were normalized to the median value of the gene to facilitate comparisons to microarray data.

### Microarray analysis

Data files were processed with GeneSpring Software (version 11.5.1; Agilent Software, Santa Clara, CA) for determination of significantly altered genes and clustering analysis. Present genes were determined from the MAS5 (Affymetrix) probe set algorithm based on a 12.5% (5/40) present call. Each sample was clustered based on MAS5 for present genes based on Pearson Correlation similarity score and average linkage clustering algorithm.

To provide a conservative choice of significantly altered genes in CP, three independent probe set algorithms were used: MAS5, RMA, and GCRMA. Requiring concordance among different probe set algorithms has recently been used as an approach to reduce false positives in data sets specific to any individual algorithm. Each probe set was normalized to the median of the microarray and then to the median of that probe set on all chips. The probe set data were then condensed into gene level data in GeneSpring by calculating the median value of all probe sets belonging to a single gene. Gene values were analyzed by 2×2 Welch ANOVA based on pathology (CP vs. Control) and muscle (gracilis vs. semitendinosus) with a Benjamini and Hochberg False Discovery Rate for multiple testing correction setting the required statistical significance to (p<0.01). For each algorithm significant genes were separated into lists for increased or decreased expression with CP, so that concordances of significant genes were all in the same direction.Accordingly, 1% of the genes deemed significant for an individual probeset algorithm are suspected to be false positives and genes that passed in all three algorithms were designated as significantly different for further analysis.

### Microarray categorical analysis

After the significantly altered genes were defined, categorical analysis was used to provide information on over represented subsets of genes. Enrichment analysis was performed on up-regulated and down-regulated genes independently with WebGausalt (http://bioinfo.vanderbilt.edu/webgestalt/) on Gene Ontology, KEGG Pathways, transcription factor targets, and microRNA targets [58], [59]. The hypergeometric statistical method was used with a Benjiman-Hochberg multiple testing correction with a significance level of (p<0.01) requiring a minimum of 3 genes per category.

Network analysis was also conducted to determine the proportion of significantly altered genes present in a recently-published muscle gene network (L. Smith, G. Meyer, and R. Lieber, submitted). As these networks are specific to skeletal muscle function, each gene in the network was also investigated in order to avoid arbitrary cutoffs. The networks were visualized using Cytoscape (Version 2.8.1; Cytoscape Consortium) [60] and the nodes colored based on expression level defined as the average expression in CP:average expression of controls. For complexes, the geometric mean of expression ratios is reported and significance is denoted if at least one gene in the complex was significantly different.

### mRNA correlations to passive stiffness

The same biopsies used for microarray analysis also underwent mechanical experiments, published separately, that allowed matching of microarray data to mechanical function [15]. Briefly, all biopsies underwent passive mechanical testing of muscle fibers and muscle fiber bundles. Fibers and bundles were isolated and stretched with the result of the mechanical tests yielding a tangent stiffness (kPa/µm sarcomere length). For fibers, this represents the stiffness of components within the cell and for fiber bundles includes cellular components as well as extracellular components. Collagen concentration was also measured in fiber bundle samples.

Correlations analysis was performed using MATLAB software (Mathworks; Natick, MA). Each gene that was considered significantly altered in children with CP from the analysis above was correlated with each of the physical parameters measured. Correlations were considered significant with p<0.05. No multiple testing correction was used in determining genes significantly correlated with mechanical data.

## Supporting Information

Table S1 **A list of each gene that was significantly altered in CP.** Gene_ID is affymetrix microarray spot ID. Ratio is expression ratio of CP to typically developing controls. P-values are listed for all 3 summarization algorithms used.
(XLSX)

Table S2 **Significantly over-represented ontologies from genes significantly up-regulated (A) or down regulated (B) in CP.** Category (C), Observed (O), Expected (E), Ratio of observed over expected (R), raw p-value (rawP), and multiple testing adjusted p-value (adjP).
(XLSX)

Table S3 **List of gene families and complexes used for** **Figure 3** **.** Complexes are separated by the functional muscle network.
(XLSX)

Table S4 **Genes significantly correlated with passive stiffness measurements on fibers and fiber bundles.**
(XLSX)

Table S5 **Primer pairs used for quantitative real-time PCR analysis.**
(XLSX)

Table S6 **Significantly over-represented ontologies from genes with positively or negatively significantly correlated with fiber or bundle passive stiffness.** Category, Observed, Expected, and multiple testing adjusted p-value (adjP).
(XLSX)

Table S7 **Similarity between upper and lower extremity transcriptional studies of skeletal muscle in ceregbral palsy.** The list of genes that are significantly regulated in the same direction with cerebral palsy between the current study in hamstring muscle and a previous study in wrist muscle. Only 13 genes were up-regulated in both and only 6 down regulated in both. The 13 up-regulated genes were significantly over-represented in the 4 extracellular matrix gene ontologies listed.
(XLSX)
